# Remote monitoring in cochlear implant users: feasibility and reliability in adolescents

**DOI:** 10.1007/s00405-026-10153-8

**Published:** 2026-03-28

**Authors:** N. M. Keijsers, H. F. E. van der Toom, A. Goedegebure, J. L. Vroegop

**Affiliations:** Department of Otorhinolaryngology and Head and Neck Surgery, Erasmus University Medical Center, Rotterdam, the Netherlands

**Keywords:** Cochlear implants, Telemedicine, Adolescents, Remote monitoring, Audiometry

## Abstract

**Purpose:**

This study evaluated the feasibility and reliability of home-based monitoring of auditory performance in adolescent cochlear implant (CI) users. Objectives were to: (1) compare self-administered Remote Check (RC) audiometry with clinic-based audiometry, (2) assess how reliably audiologists can determine follow-up needs using RC and two validated questionnaires, (3) evaluate the feasibility of an RC-based follow-up schedule and (4) explore adolescents’ and parents’ experiences with RC and their openness to replacing in-clinic visits with remote monitoring.

**Design:**

This prospective cohort study enrolled eighteen experienced CI users aged 10–19 years. Participants completed the RC test battery and two patient-reported outcome measures at home, which were reviewed by an audiologist. During annual clinical visits, aided thresholds and speech perception in noise were measured and CI fitting was assessed, which were reviewed by a second audiologist. Afterward, participants and their parents completed evaluation questionnaires.

**Results:**

Twenty-five implanted ears were included. RC-derived aided thresholds were on average 6.4 decibels lower than clinical thresholds, while digits-in-noise results did not differ significantly. Audiologists reached concordant conclusions in two-thirds of cases, while three CI related issues were not identified through RC. Questionnaire responses indicated high satisfaction, with 79% of adolescents and 88% of parents preferring RC-based follow-up.

**Conclusions:**

Most participants completed RC independently at home. Variability limits individual interpretation, and some CI issues were missed remotely. While RC shows promise as an adjunct CI aftercare for adolescents, specific limitations related to account mismatch, incomplete datalogging and adolescent bimodal users were identified.

**Supplementary Information:**

The online version contains supplementary material available at 10.1007/s00405-026-10153-8.

## Introduction

Cochlear implants (CIs) substantially improve hearing and quality of life in individuals who are hard of hearing [1], but implantation requires lifelong specialized follow-up. As candidacy criteria broaden, the number of CI users continues to grow, increasing demand for long-term care [2]. Since many adults and children reach stable auditory performance within 3–6 months after activation [3–5], routine annual appointments may be reduced by remotely monitoring stable users and scheduling clinic visits only when needed. This could free clinical resources, reduce patient travel burden, and lower environmental impact [6].

Remote Check (RC), integrated into the Nucleus Smart App by Cochlear Ltd™, enables users to complete questionnaires, impedance checks, self-administered audiometry, and speech-in-noise tests at home. Prior studies in adults and children show RC can detect clinically relevant issues and yields conclusions comparable to in-person assessments [7, 8]. However, its feasibility and clinical usefulness in adolescents and young adults remain unknown.

The aim of the present study was to investigate the feasibility of RC as a triage tool to determine if clinical follow-up is required in adolescents and young adults with CIs. Specifically, the study sought to determine whether certain follow-up appointments could be replaced by remote assessment without compromising clinical decision-making. Comparison between self-administered RC audiometry and standard clinical audiometry was conducted, together with an evaluation of the extent to which audiologists can accurately determine follow-up needs using RC data supplemented with validated patient-reported outcome measures (PROMs). In addition, the study examined how CI users and their parents perceive RC and their willingness to substitute in-clinic visits with remote monitoring.

## Methods

### Participants

This prospective cohort study was conducted at a University Medical Center and included adolescents aged nine to nineteen years attending annual CI follow-up visits between October 2024 and September 2025. Inclusion criteria were a Cochlear Ltd™ implant, aged between nine and twenty years and ≥ 1 year of CI experience. Exclusion criteria were insufficient Dutch proficiency, inadequate speech perception for the digits in noise test (DIN), or cognitive limitations preventing completion of RC or questionnaires.

### Ethics

Written informed consent was obtained from participants and, for those under 16 years, from parents or caregivers. The Medical Ethics Committee of Erasmus University Medical Center confirmed that the Medical Research Involving Human Subjects Act did not apply (MEC-2024–0074). The study adhered to the Declaration of Helsinki and GDPR.

### Study design

**Fig. 1:**
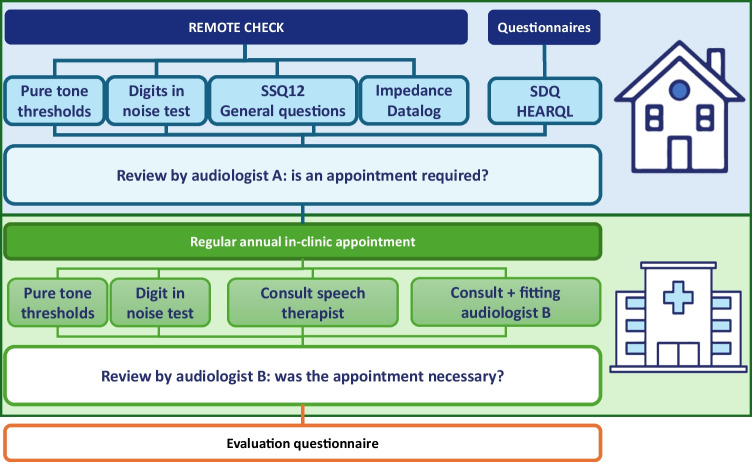
Study design overview: The first part (blue) was completed at home and included the RC test battery and two additional questionnaires, which were then reviewed by an audiologist. Afterwards, the participants attended a routine in-clinic appointment (green) for clinical audiometry, consultations with a speech therapist and an audiologist for CI fitting evaluation. This visit was reviewed by a second audiologist. Finally, the participants were given an evaluation questionnaire (orange)

An overview of the study design is shown in Fig. 1. One month before their routine appointment, participants completed the RC test battery and digital Dutch HEAR-QL and SDQ at home, with technical support if needed. An audiologist reviewed all RC data, including medical history, and documented whether an in-clinic visit would have been recommended.

All participants, irrespective of the audiologist’s preliminary assessment, then underwent a standard in-clinic CI checkup, including clinical PTA and DIN testing, followed by speech therapist and audiologist evaluation and device adjustments when required. To ensure consistency with RC data, testing was limited to the implanted ear for bimodal users, while both ears were assessed separately in bilateral CI users. When necessary, the contralateral ear was masked in accordance with standard clinical protocols.

The second audiologist independently judged whether the visit had been necessary. Audiologists assessing RC and in-clinic data were different and blinded to each other’s conclusions.

After the visit, adolescents and parents completed brief custom RC experience questionnaires. This.

### Materials

RC is integrated in the Nucleus Smart App and developed by the CI manufacturer Cochlear Ltd™. A detailed description of RC has been published by Maruthurkkara et al.[7]. Implant-site photographs were omitted to improve feasibility [11]. Participants also completed Dutch versions of the Hearing Environments And Reflection on Quality of Life questionnaire (HEAR-QL) [9], and the Strengths and Difficulties Questionnaire (SDQ) [10], which help assess broader well-being. Both PROMs are included in the standard of care pathway during annual CI follow-up appointments in the participating clinic.

After the clinical visit, participants completed a brief custom evaluation form assessing the overall experience with RC and openness to an RC-based follow-up schedule. The questionnaire is added to Supplemental Digital Content 1.

### Data management and analysis

Pseudo-anonymized data was stored in Castor EDC. RC data was additionally stored automatically in Cochlear Ltd™’s clinical portal for review.

Analyses were performed in RStudio build 513. Non-normality was indicated using the Shapiro–Wilk test Differences between the clinical and RC DIN and PTA results were analyzed using a paired Wilcoxon Signed Ranked Test with Bonferroni correction. Questionnaire data were analyzed quantitatively and qualitatively.

## Results

### Participants

Eighteen participants were enrolled, of which nine were female and nine were male. The mean age was 14.5 years. CI experience ranged from 4 to 17 years, with an average of ten years. Eleven participants were unilateral CI users and seven were bilateral CI users, resulting in a total of 25 CIs. Four unilateral users wore a contralateral hearing aid. The demographics are shown in Table 1.

**Table 1:** demographics of included participants. In case of subsequently implanted bilateral users, the years of CI use is based on the date of first implantation. For sequentially implanted bilateral CI users, the years of CI use are calculated based on the first implantation

		*N*
*Gender*		
	Male	9
	Female	9
*CI’s*		
	Unilateral	7
	Bilateral	7
	Bimodal	4
*Age (years)*		
	Average	14.5
	Range	10–19
*Years of CI use (years)*		
	Average	10
	Range	4–17
*Implant model*		
	CI24RE	9
	CI512	10
	CI612	4
	CI632	2

Datalogs were the most frequently missing component in RC data, which was unavailable in seven (39%) participants. Two bilateral users provided data from only one side, due to smartphone limitations or loss of a processor. Other missing data includes lack of RC audiometry due to technical limitations of the smartphone (n = 1) and missing DIN results due to uncompleted practice runs (n = 1).

### Audiometry comparison

**Fig. 2:**
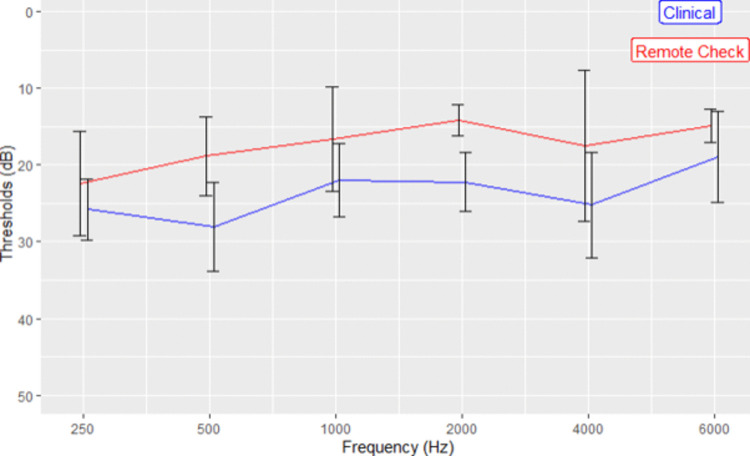
Comparison of the means thres holds measured at home through RC (blue) and in clinic (red). The error bars show the SD. While measurements are horizontally slightly offset from each other to enhance the visibility of the individual error bars, the RC and clinical measurements were obtained for the exact same frequencies

**Fig. 3:**
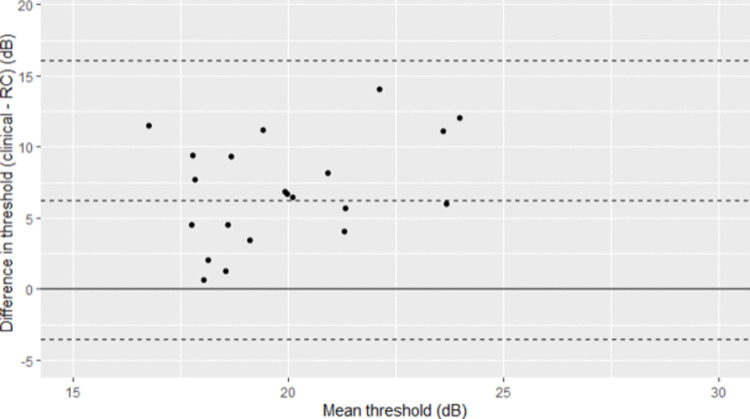
Bland–Altman plot of measured PTA’S. The X-axis shows the mean threshold averaged across RC and clinically measurements, whereas the Y- axis shows the difference between the measurements (clinical minus RC).For each participants

Twenty-two paired aided PTA measurements were obtained. Mean clinical PTA was 23.9 dB HL (Standard deviation (SD) 6.2) and RC PTA 17.5 dB HL (SD 6.7). A significant mean difference (clinical minus RC) of 6.4 dB (p < 0.01), indicating consistently lower RC thresholds. Audiometry results are also shown in Fig. 2 and Supplemental Digital Content 2. Frequency-specific differences were significant across all frequencies. Bland–Altman analysis revealed systematically lower RC thresholds. The results are shown in Fig. 3.

**Fig. 4:**
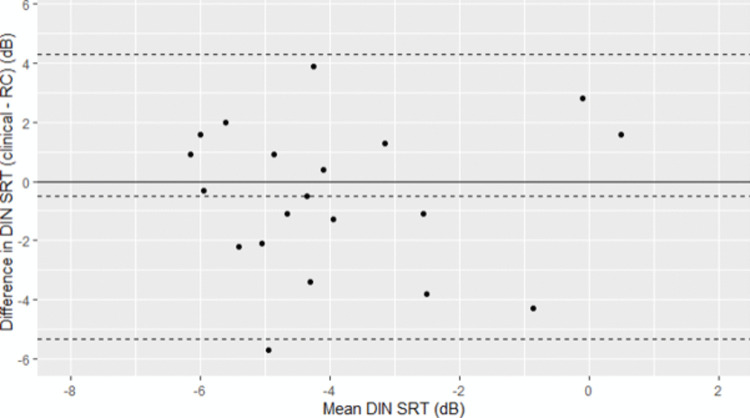
Bland–Altman plot of the DIN. The X-axis shows the mean DIN results for RC and clinically obtained thresholds combined, whereas the Y-axis shows the difference between the measurements. Difference are calculated as clinical DIN minus RC DIN. The full line at 0 indicates no difference and the middle dash line shows the mean difference. The lower and upper dash line represent the lower and upper limits of agreement respectively

DIN data was available from 18 ears. Mean clinical SRT was –4.2 dB (SD 2.2) and RC SRT –3.7 dB (SD 2.2), with no significant difference (p = 0.45). A Bland–Altman plot illustrating the agreement between clinical and RC-based DIN measurements is presented in Fig. 4, showing most differences fell within ± 2 dB.

### Potential issue detection

RC and clinical assessments yielded concordant conclusions in 12/18 cases (67%). Two RC-based recommendations for clinical follow-up were false positives as reported issues could have been managed remotely. Four clinically necessary follow-ups (22%) were not identified through RC, including CI mapping optimization (n = 2), hearing aid adjustment (n = 1), and speech therapy referral (n = 1). The results are presented in Table 2A.

**Table 2:** Overview of audiologist reviews. The columns show the number of participants the audiologist would have called in for appointment based on RC, while the rows show whether the clinical appointment was considered necessary. Table 2A shows the results of all participants, while bimodal users are excluded in Table 2B. *includes two participants who did not provide a completed RC, review is based on the other information provided

2A: all participants		Is appointment needed, based on RC?		
		Yes	No	Total
Was clinical appointment necessary?	Yes	2	4	6
	No	2	10*	12
	Total	4	14	
2B: Exclusively CI users (unilateral or bilateral), bimodal users excluded		Is appointment needed, based on RC?		
		Yes	No	Total
Was clinical appointment necessary?	Yes	2	2	4
	No	2	8*	10
	Total	4	10	

Four participants were bimodal users. As RC does not capture any data from the contralateral hearing aid, the audiologist lacked information. Table 2B shows the results of the reviews when these four bimodal users are excluded. In this scenario, concordance increased to 71%.

### User experience

Fourteen participants (78%) and ten parents (91%) returned evaluation questionnaires. The results are shown in Table 3. All but one participant successfully completed the full RC test battery, and 86% reported RC as easy to use. Amongst participants under 18 years old, 60% completed RC and the questionnaires without parental assistance. Thirty-six percent of participants and half of parents valued annual audiologist contact, though some found phone contact sufficient. Seventy-nine percent of participants and 88% of parents reported willingness to adopt an RC-based follow-up schedule. Open comments included RC audiometry feeling easier or longer, and isolated reports of loud DIN stimuli during RC.

**Table 3:** results of evaluation questionnaire from both participants and the parents of parents younger than 18 years. For a better overview, the multiple-choice options very easy/positive/difficult/negative were grouped together with easy/positive/difficult/negative. The percentage of the total answers for those questions is shown between brackets. Some parents left a few questions open as their child completed RC by themselves

Question topic	Response options	Participants (n = 14)	Parents (n = 10)
Ease of use RC	Easy	12 (86%)	6 (75%)
	Neutral	0	1 (13%)
	Difficult	1 (7%)	0
	Unable to complete	1 (7%)	1 (13%)
Ease of filling in the additional questionnaires	Easy	12 (86%)	7 (88%)
	Neutral	2 (14%)	1 (13%)
	Difficult	0	0
	Unable to complete	0	0
Assisted child with RC and questionnaires	Only RC	N/A	1 (10%)
	Only questionnaires	N/A	1 (10%)
	Both	N/A	2 (20%)
	Neither	N/A	6 (60%)
Opinion on RC	Positive	11 (79%)	8 (88%)
	Neutral	2 (14%)	1 (11%)
	Negative	1 (7%)	0
Was RC audiometry comparable to clinical	Different, prefer clinic	5 (36%)	N/A
	Different, prefer RC	3 (21%)	N/A
	Similar	6 (43%)	N/A
Annual contact with audiologist	Important	5 (36%)	5 (50%)
	Neutral	6 (43%)	3 (30%)
	Not very important	3 (21%)	2 (20%)
Replace periodic follow-up with RC-based follow-up	Yes	11 (79%)	8 (88%)
	Neutral	3 (21%)	1 (11%)
	No	0	0

## Discussion

The present study evaluated the feasibility of remote monitoring using RC as an alternative to traditional annual in-clinic follow-up for adolescent and young adult CI users. Findings indicate that RC could be a valuable addition to CI aftercare pathways for adolescent CI users with stable performance. Differences between RC-based and clinically obtained audiometry were largely consistent with previous literature, whereas accuracy of issue detection was lower than reported in adult CI users. Nonetheless, adolescents were receptive to an RC-based follow-up schedule.

### Audiometry comparison

Differences in PTA results between RC and clinical measurements were comparable to literature in adults. RC thresholds were, on average, 6.4 dB lower than clinical thresholds, similar to the 6.7 dB difference reported by Marutkhurkkara et al. [7]. This may be partly attributed to methodological differences and to delivery pathway effects, as other connectivity-based comparisons have also shown lower thresholds. [12]. While mean differences were clinically acceptable, substantial individual variability warrants caution. Establishing a baseline RC measurement is likely to provide a more valid reference point than direct comparisons with clinical audiometry. DIN results showed no significant difference, unlike earlier reports [7].

### Potential issue detection

Previous studies, encompassing 153 adults and 20 children, reported 99% concordance between RC-based and in-clinic assessments [7, 8], whereas the present study observed only 67%. One potential explanation for this difference is that, unlike in previous studies, the clinical follow-up audiologist in the present study was blinded to the RC results. Another factor may be the inclusion of bimodal users. Half of the missed issues involved bimodal users, including one related to hearing aid fitting. As RC does not capture hearing aid data, the reviewing audiologist lacked complete information for decision-making. Although earlier work reported high detection rates in adults, including bimodal users, insufficient subgroup data prevent meaningful comparison [7]. However, it is plausible that the bimodal users in those studies had poorer contralateral hearing aid performance than those in the present sample, all of whom achieved 80–100% speech perception in quiet. This difference in hearing aid performance may explain the discrepancy in findings. Importantly, because Remote Check does not capture contralateral hearing-aid data, adolescent bimodal users may be less suitable for exclusively RC-based follow-up at this time.

During this study, three CI-related issues were missed through Remote Check. These issues were also not detectable retrospectively due to stable audiometry and minimal concern expressed by participants. However, one participant showed a reduced SDQ score, although insufficient to trigger an appointment. Combined with the finding that a reduced PROM score prompted follow-up for another participant, this highlights the added value of including validated PROMs in the RC battery. Clinicians should be aware that not all issues in adolescent CI users can be detected through RC alone.

### User experience

Most adolescents aged over ten successfully completed RC. Participants who could not complete RC were constrained by technical limitations rather than task complexity. As in previous studies[7, 13], acceptance was high, with 79% of adolescents and 88% of caregivers supporting RC-based follow-up. Some participants encountered technical issues, mostly related to RC not appearing in the Nucleus Smart App due to account mismatches, reflecting the transition from parent-managed to self-managed accounts. Other issues involved device pairing or smartphone limitations.

Furthermore, datalogs were unavailable in RC for half the participants, despite being accessible from the processor in the clinic. This was later traced to infrequent Nucleus Smart App use, which is required for datalog transfer. This is noteworthy, as reliable datalogging is particularly important in this age group, as literature shows 3.8–15.2% inconsistent or discontinued CI use [14].

### Limitations

The single-center feasibility study was limited by sample size and inclusion of only one manufacturer’s RC platform. Both participating audiologists were inexperienced users of RC, which shows that clinicians can relatively easily and rapidly adopt RC. However, decision-making may have differed if the audiologists possessed prior RC experience.

## Conclusion

Most adolescents can complete RC successfully. Overall, thresholds obtained through RC were consistently lower, whereas speech in noise test results were comparable. Nevertheless, given the observed individual variability, a baseline RC assessment should be obtained before transitioning adolescents to RC-based follow-up. Not all CI-related issues were detectable through RC, but RC may serve as supplementary triage tool for adolescents with stable CI performance and potentially reduce routine clinical workload. This study identified specific considerations for adolescents regarding account setup, datalogging, and bimodal use that audiologists should consider during RC-based follow-up candidate selection. Acceptance among adolescents and caregivers is high, supporting broader integration of RC into clinical practice.

Future work should identify predictors of successful RC-based monitoring and evaluate whether brief supplemental remote interactions can enhance issue detection while preserving the personal contact valued by families.

## Supplementary Information

Below is the link to the electronic supplementary material.

Supplementary file1 (PDF 163 KB)

Supplementary file2 (PDF 87 KB)

Supplementary file3 (PDF 52 KB)
