# Supplementary material for: Remote monitoring in cochlear implant users: feasibility and reliability in adolescents
Source: Eur Arch Otorhinolaryngol. 2026 Mar 28;283(6):4009–15. doi: 10.1007/s00405-026-10153-8 (PMC13249620; doi:10.1007/s00405-026-10153-8)
Supplement: Supplementary file 1 — Supplementary file1 (PDF 163 KB) [file 405_2026_10153_MOESM1_ESM.pdf]

Supplemental Digital Content 1: evaluation questionnaire for participants and parents (these questionnaires are translated to English for publication, but only the Dutch version was used during the study)

Questions about your experience with measuring your own hearing at home

This questionnaire has a front and a back.

1. How difficult / easy was it to measure your own hearing through Remote Check?

O Did not succeed
O Very difficult
O Difficult
O Neutral
O Easy
O Very easy

2. How difficult / easy was it to fill in the questionnaires digitally at home?

O Did not succeed
O Very difficult
O Difficult
O Neutral
O Easy
O Very easy

3. How did you experience measuring your own hearing through Remote Check?

O Very unpleasant
O Unpleasant
O Neutral
O Positive
O Very positive

4. Were the hearing tests in Remote Check comparable to the hearing tests you do at the clinic?

O It was different and I prefer to do them in the clinic
O It was different and I prefer to do it at home
O It was comparable

5. Would you like to use Remote Check more often in the future?

O Definitely not
O Preferably not
O Neutral
O Probably
O Definitely

6. How important do you rate the annual contact with your audiologist, even if you have no questions or complaints about your hearing?

O unimportant
O Not very important
O Neutral
O Important
O Very important

7. Imagine that you will get fewer appointments in the hospital for your hearing in the future. Instead, every now and then you measure your own hearing through Remote Check. If your score on Remote Check is good, you don't get an appointment in the clinic that year. Would you be interested in this?

O Definitely not
O Preferably not
O Neutral
O Probably
O Definitely

8. Do you have any questions or comments?

……

……

……

……

……

……

Questions about your experience with measuring your child’s hearing at home

This questionnaire has a front and a back.

1. Did you help your child with completing the hearing tests or questionnaires at home?
   O No, my child did everything by themselves
   O Yes, I helped with completing the hearing tests
   O Yes, I helped with filling in the questionnaires
   O Yes, I helped with both completing the hearing tests and filling in the questionnaires

2. How difficult / easy was it to measure your child’s hearing at home?

   O Did not succeed
   O Very difficult
   O Difficult
   O Neutral
   O Easy
   O Very easy

3. How difficult / easy was it to fill in the questionnaires digitally at home?

   O Did not succeed
   O Very difficult
   O Difficult
   O Neutral
   O Easy
   O Very easy

4. How did you experience measuring your child's hearing through Remote Check?

   O Very unpleasant
   O Unpleasant
   O Neutral
   O Positive
   O Very positive

5. Would you like your child to use Remote Check more often in the future?

O Definitely not
O Preferably not
O Neutral
O Probably
O Definitely

6. How important do you rate the annual contact with your child's audiologist, even if you or your child have no questions or complaints about your hearing?

O unimportant
O Not very important
O Neutral
O Important
O Very important

7. Imagine that your child will get fewer appointments in the hospital for their hearing in the future. Instead, every now and then they measure their own hearing through Remote Check. If your score on Remote Check is good, you don't get an appointment in the clinic that year. Would you be interested in this?

O Definitely not
O Preferably not
O Neutral
O Probably
O Definitely

8. Do you have any questions or comments?

....

....

....

....

....
