# Supplementary material for: Remote monitoring in cochlear implant users: feasibility and reliability in adolescents
Source: Eur Arch Otorhinolaryngol. 2026 Mar 28;283(6):4009–15. doi: 10.1007/s00405-026-10153-8 (PMC13249620; doi:10.1007/s00405-026-10153-8)
Supplement: Supplementary file 2 — Supplementary file2 (PDF 87 KB) [file 405_2026_10153_MOESM2_ESM.pdf]

Supplemental Digital Content 2: overview of mean thresholds for both clinical and RC measurements.

The difference, calculated as clinical minus RC, and the returned p-value of the paired Wilcoxon-Signed Rank test with Bonferroni correction are also shown.

Frequency in Hz	Method	Mean (SD) in dB	Difference (p value) in dB
**250**	Clinical	25.9 (4.3)	3.5 (p=0.044)
	RC	22.4 (6.8)	
**500**	Clinical	28.4 (4.7)	9.6 (p<0.01)
	RC	18.9 (5.2)	
**1000**	Clinical	22.3 (3.1)	5.6 (p<0.01)
	RC	16.7 (6.8)	
**2000**	Clinical	22.3 (2.9)	8.1 (p<0.01)
	RC	14.2 (2.1)	
**4000**	Clinical	25.5 (6.4)	8.0 (p<0.01)
	RC	17.5 (9.8)	
**6000**	Clinical	19.3 (6.0)	4.4 (p=0.049)
	RC	14.8 (2.2)	
