# Supplementary material for: Remote monitoring in cochlear implant users: feasibility and reliability in adolescents
Source: Eur Arch Otorhinolaryngol. 2026 Mar 28;283(6):4009–15. doi: 10.1007/s00405-026-10153-8 (PMC13249620; doi:10.1007/s00405-026-10153-8)
Supplement: Supplementary file 3 — Supplementary file3 (PDF 52 KB) [file 405_2026_10153_MOESM3_ESM.pdf]

Supplemental Digital Content 3: overview of reviews by the audiologist.

			n
**Remote check**	Is appointment recommended based on Remote Check	Yes	4
		No	14
	Is appointment recommended based on Remote Check and questionnaires?	Yes	4
		No	14
**Clinic**	Where changes made to the CI processor?	Small changes	6
		Large changes	3
		Changes to configuration	1
		None	8
	Was appointment with audiologist necessary?	Yes	5
		No	13
	Was appointment with speech therapist necessary?	Yes	2
		No	16
